# In Situ High Selectivity Contact‐Electroreduction of CO_2_ to Methanol Using an Imine‐Mediated Metal‐Free Vitrimer Catalyst

**DOI:** 10.1002/anie.202500222

**Published:** 2025-03-22

**Authors:** Nannan Wang, Haisong Feng, Jing Yang, Jie Zheng, Yong‐Wei Zhang, Nikos Hadjichristidis, Zibiao Li

**Affiliations:** ^1^ Institute of Sustainability for Chemicals Energy and Environment (ISCE2) Agency for Science Technology and Research (A*STAR) 1 Pesek Road, Jurong Island Singapore 627833 Republic of Singapore; ^2^ State Key Laboratory of Chemical Resource Engineering Beijing Advanced Innovation Center for Soft Matter Science and Engineering Beijing University of Chemical Technology Beijing 100029 P.R. China; ^3^ Institute of High Performance Computing (IHPC) Agency for Science Technology and Research (A*STAR) 1 Fusionopolis Way, #16‐16 Connexis Singapore 138632 Republic of Singapore; ^4^ Polymer Synthesis Laboratory KAUST Catalysis Center Physical Science and Engineering Division King Abdullah University of Science and Technology (KAUST) Thuwal 23955 Saudi Arabia; ^5^ Institute of Materials Research and Engineering (IMRE) Agency for Science Technology and Research (A*STAR) 2 Fusionopolis Way, Innovis #08‐03 Singapore 138634 Republic of Singapore; ^6^ Department of Materials Science and Engineering National University of Singapore 9 Engineering Drive 1 Singapore 117576 Singapore

**Keywords:** CO_2_ reduction, Contact‐electrocatalysis, High‐selectivity, Metal‐free catalyst, Vitrimer

## Abstract

Metal catalysts for the CO_2_ reduction reaction (CO_2_RR) face challenges such as high cost, limited durability, and environmental impact. Although various structurally diverse and functional metal‐free catalysts have been developed, they often suffer from slow kinetics, low selectivity, and nonrecyclability, significantly limiting their practical applications. In this study, we introduce a recyclable nonmetallic polymer material (vitrimer) as a catalyst for a new platform in contact‐electrocatalysis. This approach harnesses the contact charges generated between water droplets and vitrimer to drive CO_2_RR, achieving methanol selectivity exceeding 90%. The imine groups within the vitrimer play a dual role, facilitating CO_2_ adsorption and enriching friction‐generated electrons, thereby mediating efficient electron transfer between the imine groups and CO_2_ to promote CO_2_RR. After 84 h of CO_2_RR, the system achieved a methanol production rate of 13 nmol·h^−1^, demonstrating the excellent stability of the method. Moreover, the vitrimer retains its high‐performance electrocatalytic activity even after recycling. Mechanistic studies reveal that, compared to traditional metal catalysts, the N─O bond in the imine, which adsorbs the key intermediate *OCH_3_, breaks more readily to produce methanol, resulting in enhanced product selectivity and yield. This efficient and environmentally friendly contact‐electroreduction strategy for CO_2_ offers a promising pathway toward a circular carbon economy by leveraging natural water droplet‐based contact‐electrochemistry.

## Graphical abstract

A vitrimer catalyst with C═N bond enables efficient CO_2_ reduction via contact‐electrocatalysis, driven by water droplet‐induced charges. Imine groups stabilize CO_2_ adsorption, enrich electrons, and mediate electron transfer, achieving >90% methanol selectivity. The system demonstrates exceptional stability, recyclability, and sustained catalysis, offering a sustainable pathway for CO_2_ conversion to methanol.

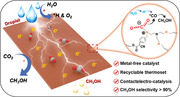

## Introduction

The conversion of CO_2_ into value‐added chemicals and fuels represents one of the most promising strategies to address pressing energy and environmental challenges.^[^ ^1^, ^2^, ^3^ ^]^ In recent years, extensive studies have focused on reducing CO_2_ in various products using advanced electrocatalysis, photocatalysis, and piezocatalysis methods. Notably, electrochemical reduction of CO_2_ powered by renewable sources, such as triboelectricity generated through mechanical energy, offers a potential pathway for CO_2_ transformation under mild conditions.^[^ ^4^, ^5^ ^]^ Unlike traditional electrocatalysis, which requires the formation of an electric current loop, the contact‐electrocatalytic process relies on the electrostatic charges (electrons) generated by friction to directly participate in the catalytic process in situ, eliminating the need for a conductive catalyst. Among the possible products, alcohols with high energy density, particularly methanol, are highly desirable.^[^ ^6^, ^7^, ^8^ ^]^ As a vital platform molecule in the chemical industry, methanol holds significant potential as a direct, clean fuel. However, the electroreduction of CO_2_ to methanol presents inherent challenges due to the complex six‐electron transfer pathway and slow kinetics involved.^[^ ^9^ ^]^ Metal‐based catalysts exhibit excellent catalytic activity and selectivity in the reduction of CO₂ to methanol.^[^ ^10^, ^11^ ^]^ Many transition metals, such as Cu, Pd, and In, have been extensively studied and proven to effectively facilitate the reorganization of C─H and C─O bonds, thereby enhancing the faradaic efficiency and yield of methanol. Benefiting from their tunable electronic structures and stable catalytic centers, metal‐based systems demonstrate high conversion efficiency in practical applications. However, metal‐based catalysts often rely on precious or rare metals, which are costly and scarce, posing challenges that hinder their large‐scale commercialization in renewable energy technologies. Additionally, hybrid structures' interfacial complexity and thermodynamic instability often lead metal‐based catalysts to suffer from low selectivity, poor durability, and susceptibility to gas poisoning and environmental degradation.^[^ ^12^ ^]^ Consequently, developing low‐cost, efficient alternatives to metal‐based catalysts is imperative for advancing sustainable energy solutions.

Metal‐free catalysts have gained increasing attention as efficient electrocatalysts for CO_2_RR due to their low cost and high catalytic activity, demonstrating electrocatalytic performance that rivals that of precious metal catalysts.^[^ ^13^, ^14^, ^15^ ^]^ However, despite these advancements, the synthesis process of nonmetal catalysts remains highly complex. Moreover, their CO_2_RR performance is still largely constrained by slow kinetics, high overpotentials, and lower selectivity compared to their metal‐based counterparts. A critical limitation of most nonmetallic catalysts, such as carbon nanotubes (CNTs), covalent–organic frameworks (COFs), and hexagonal boron nitride (h‐BN), is their nonrecyclability. The disposal of these materials as waste poses severe environmental risks, potentially creating new ecological challenges in the effort to mitigate CO_2_ pollution. Furthermore, the high costs associated with managing this waste add an additional layer of complexity to the widespread adoption of nonmetallic catalysts.

Due to the strong electron affinity of the C═N bond, imine‐based metal‐free catalysts facilitate efficient CO₂ adsorption and promote the formation of key reduction intermediates, such as *COOH and *CO. These properties have made imine‐based electrocatalysts a prominent focus of research efforts.^[^ ^16^, ^17^ ^]^ Furthermore, imine bonds exhibit dynamic characteristics through imine exchange or metathesis reactions, enabling the development of covalent adaptable networks (CANs). CANs, which feature rearrangeable topologies, represent a new class of materials that bridge the gap between high‐strength, durable thermosets and flexible, reprocessable thermoplastics, garnering increasing attention in recent years.^[^ ^18^, ^19^ ^]^ Within the realm of CANs, “vitrimers” have emerged as a standout innovation, utilizing an associative bond exchange mechanism.^[^ ^20^ ^]^ This mechanism preserves a consistent cross‐linking density during processing, ensuring excellent dimensional stability and enhanced material properties.^[^ ^21^, ^22^ ^]^ However, imine‐based vitrimers often rely on active nucleophilic amino groups or catalysts, which can trigger undesirable side reactions and compromise performance. To address these challenges, we recently introduced a novel metathesis reaction between C═C and C═N bonds to fabricate high‐performance vitrimers.^[^ ^23^ ^]^ This innovative approach offers several advantages over traditional imine exchange or metathesis reactions, including higher efficiency, catalyst‐free operation, and exceptional moisture resistance. These features position the C═C/C═N metathesis reaction as a groundbreaking tool to advance vitrimer technology and unlock new possibilities in the design of next‐generation materials.

Herein, we propose, for the first time, the use of a covalent adaptable network (i.e., vitrimer) featuring both C═C and C═N bonds as a nonmetal catalyst for in situ solid–liquid contact‐electrocatalytic CO_2_RR to produce methanol. The strong adsorption capability of the imine bonds within the vitrimer stabilizes the adsorption configuration of CO_2_, thereby lowering its activation energy and promoting the formation of the *COOH intermediate. Furthermore, the imine groups effectively enrich frictional charges (electrons) generated by the impact of water droplets on vitrimer and mediate the electron transfer from C═N bond to CO_2_, further facilitating the formation of reaction intermediates. This synergy enhances both the efficiency and selectivity of CO_2_ conversion. The results demonstrate that the contact‐electrocatalytic CO_2_RR sustains catalysis for up to 84 h with remarkable stability, achieving a methanol production rate of 13 nmol·h^−1^ and an outstanding selectivity exceeding 90%. Moreover, the presence of both C═C and C═N bonds within the polymer network enables excellent recyclability through the C═C/C═N metathesis reaction. More importantly, the methanol production rate from the vitrimer‐based contact‐electrocatalytic CO_2_RR remains virtually unchanged before and after recycling, demonstrating the reliability of this method and its promising potential for practical applications. This vitrimer‐based contact‐electroreduction strategy, utilizing natural water droplets, not only offers a viable pathway for methanol production but also opens new avenues for designing advanced catalysts that combine high performance, recyclability, and environmental compatibility.

## Results and Discussion

### Preparation and Characteristics of the CCCN‐Vitrimer

The dynamic properties of a vitrimer containing C═C/C═N bonds (CCCN‐vitrimer) was prepared by mixing commercially available terephthalaldehyde (TPA), tris(2‐aminoethyl) amine (TAA), and a bifunctional cross‐linker (2AC) in a one‐pot, catalyst‐free process (Figure S1). ^1^H NMR spectra of the cross‐linker (2AC) are shown in Figure S2. In the design of CCCN‐vitrimer, electron‐withdrawing functional groups, such as cyano and carbonyl groups (Figure S3), were intentionally introduced to enhance the triboelectric charge storage performance of the vitrimer.^[^ ^24^ ^]^ The FTIR spectra of the monomers (2AC, TPA, and TAA) and the CCCN‐vitrimer are shown in Figure S4. Compared to the monomers, the FTIR spectrum of CCCN‐vitrimer exhibits two distinct absorption peaks at approximately 1601 and 1639 cm⁻¹, corresponding to the C═C and C═N bonds, respectively, confirming the formation of the cross‐linked network containing these bonds.

To further validate the formation of the cross‐linked polymer, we conducted swelling tests on CCCN‐vitrimer in various solvents, including dimethylformamide (DMF), dichloromethane (DCM), tetrahydrofuran (THF), acetonitrile (ACN), ethanol (EtOH), and H₂O. The CCCN‐vitrimer network demonstrated high stability, exhibiting low swelling ratios in solvents such as DCM (28%), ACN (11%), and EtOH (5%), indicative of a highly cross‐linked structure (Figures S5,S6). Additionally, gel content analysis in these solvents (DMF, DCM, THF, ACN, EtOH, and H₂O) revealed high gel content (≥95%) across all samples (Figure S5), further supporting the formation of the cross‐linked network.

As shown in Figure S7, the initial decomposition temperature (*T*_5%_, temperature at 5% weight loss) of CCCN‐vitrimer is approximately 270 °C, indicating excellent thermal stability. The glass transition temperature (*T*_g_) of CCCN‐vitrimer was found to be 121 °C as determined by differential scanning calorimetry (DSC) (Figure S8), slightly lower than the 136 °C obtained by dynamic mechanical analysis (DMA) (Figure S9), due to differences in measurement techniques. The storage modulus of CCCN‐vitrimer as a function of temperature shows a high value of 1445 MPa below *T*_g_ (136 °C) and a robust rubbery plateau of 35.06 MPa above *T*_g_, indicating a well‐defined cross‐linked structure (Figure S9). The cross‐linking density (*ρ*) of the polymer network was calculated to be 3127 mol m^−3^. Such a high cross‐linking density imparts increased rigidity and enhanced dimensional stability, making the material exceptionally durable and well‐suited for real‐world applications.

**Figure 1:**
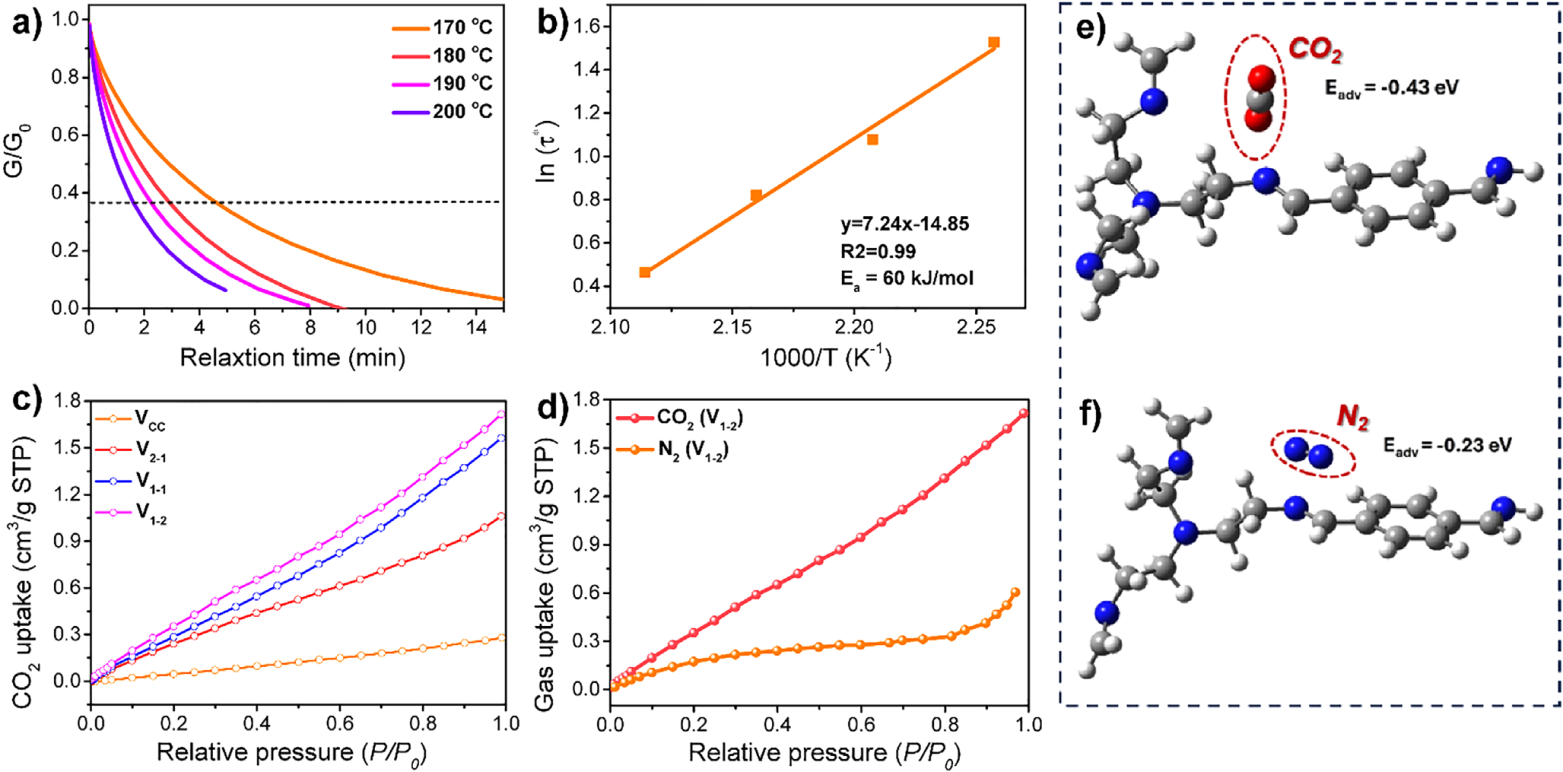
Preparation and characteristics of the CCCN‐vitrimer. a) Stress relaxation curves of CCCN‐vitrimer at different temperatures. b) Fitting of the relaxation time to the Arrhenius equation. c) CO_2_‐BET of vitrimers with different imine contents. d) CO_2_‐ and N_2_‐BET of CCCN‐vitrimer. e,f) Comparison of the adsorption energies (*E*_adv_) of CO_2_ and N_2_ on CCCN‐vitrimer.

Due to the presence of both C═C and C═N bonds within the polymer network, the resulting CCCN‐vitrimer exhibits a rearrangeable topology through C═C/C═N metathesis (Figure S10). The dynamic feature of C═C/C═N exchange within the polymer network was analyzed via stress relaxation at elevated temperatures (170, 180, 190, and 200 °C) using DMA. As shown in Figure 1a, the stress relaxation moduli decreased exponentially over time, indicating that the cross‐linked polymer behaves as a vitrimer, capable of stress relaxation over extended periods due to the dynamic C═C/C═N exchange reaction. The relaxation time (τ*), defined as the time required to relax 1/*e* (36.7%) of the initial stress, was determined using the Maxwell model for viscoelastic fluids. With increasing temperature, the exchange rate accelerated, and τ* decreased from 4.59 min at 170 °C to 1.61 min at 200 °C. According to Arrhenius’ law, the activation energy (*E*_a_) for the C═C/C═N exchange reaction in CCCN‐vitrimer was calculated to be 60 kJ mol⁻¹ (Figure 1b), consistent with results for other materials containing C═C/C═N bonds.^[^ ^23^ ^]^ Thanks to these dynamic C═C/C═N linkages, CCCN‐vitrimer exhibits efficient reprocessing capabilities. After mechanical failure, the CCCN‐vitrimer sample was cut into small pieces and hot‐pressed at 180 °C and 30 bar for 30 min, completely restoring the polymer network (Figure S11). The recycled CCCN‐vitrimer retained its surface properties, as evidenced by contact angle measurements of 93.93° before recycling and 94.23° after recycling (Figure S12). Furthermore, FTIR analysis confirmed that no significant structural changes occurred during reprocessing (Figure S13).

Given that imine‐modified catalysts are commonly used to enhance CO₂ adsorption (Table S1), the primary role of introducing C═N groups into the vitrimer is to increase CO₂ adsorption capacity, thereby facilitating the subsequent contact electroreduction of CO_2_. We then characterized the CO_2_ adsorption capacity of CCCN‐vitrimer with varying C═N bond content, as shown in Figure 1c. Based on the ratio of C═C to C═N bonds, CCCN‐vitrimers with different imine contents were prepared and designated as V_2‐1_, V_1‐1_, and V_1‐2_ in order of increasing imine content. A control vitrimer without C═N bonds was also synthesized and designated vitrimer‐CC (Figures S14,S15). The results indicate that CO₂ adsorption increases with higher C═N content, demonstrating that the incorporation of imine groups effectively enhances the CO_2_ capture capacity of CCCN‐vitrimer. Importantly, imine groups selectively adsorb CO_2_, whereas their adsorption of N_2_ remains very weak (Figure 1d), highlighting the practical applicability of imine‐functionalized vitrimers for CO_2_ capture under real‐world conditions. To further investigate CO_2_ adsorption, DFT calculations were performed to determine the binding energies of CO_2_ and N_2_ with the vitrimer. The results revealed that the adsorption energy between CCCN‐vitrimer and CO_2_ molecules is −0.43 eV (Figure 1e), whereas for N₂ is −0.23 eV (Figure 1f). These findings confirm that CO_2_ is preferentially and more readily adsorbed onto the CCCN‐vitrimer molecules, consistent with the experimental results.

### Device and Catalytic Process for Contact‐Electroreduction of CO_2_

**Figure 2:**
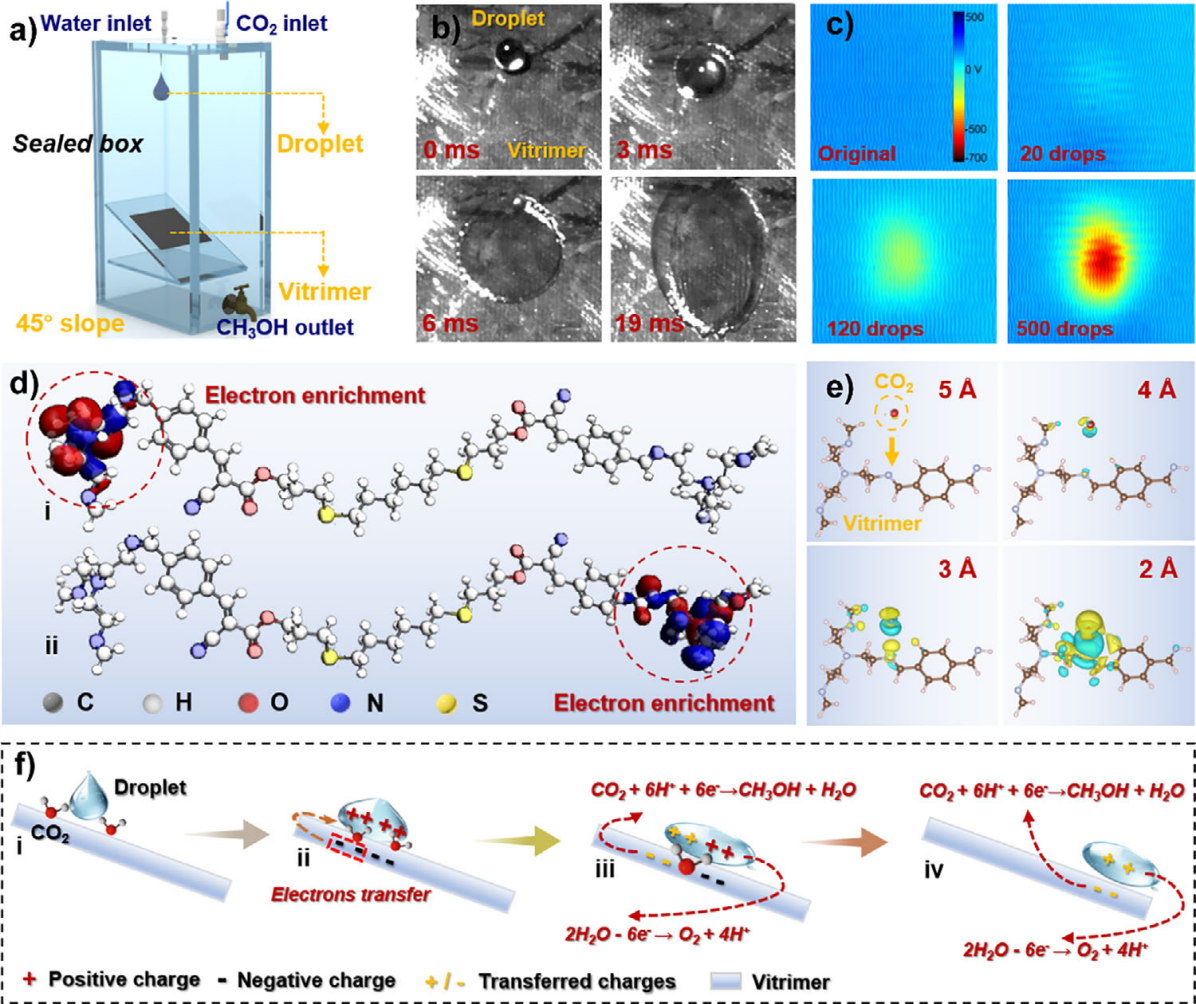
Device and catalytic process for the contact‐electroreduction of CO_2_. a) Schematic diagram of the contact‐electrocatalytic device for methanol production. b) Dynamic optical image showing the water droplets spreading on the vitrimer surface. c) Effect of water droplet impact number on surface potential distribution. d) Electron distribution of the conduction band edge in the CCCN‐vitrimer. e) Charge distribution during the contact between CO_2_ molecules and vitrimer. f) Schematic representation of the integrated charge generation and consumption process in the water droplet–vitrimer contact‐electrocatalytic CO_2_RR.

Building on the aforementioned foundational study, we incorporated the prepared CCCN‐vitrimer into a solid–liquid contact electrification system to develop a metal‐free contact‐electroreduction of CO_2_ device. The schematic of the device used for methanol production via contact‐electroreduction of CO₂ is shown in Figure 2a. Inside a 2.5 L sealed box, a 45‐degree inclined plane coated with the vitrimer is positioned 30 cm below the water inlet. Adjacent to the water inlet is a CO_2_ inlet, which also serves as the outlet for CO_2_ gas products. A reservoir at the bottom of the chamber collects methanol as the primary liquid product. During operation, the generated methanol is enriched through circulation using a small peristaltic pump, allowing for more precise measurement of product yield. A photograph of the complete setup is provided in Figure S16. Firstly, the surface potential of different vitrimers upon water droplet impact was compared, as shown in Figure S17. The results indicate that the surface potential of the vitrimers decreases with the reduction in the content of cyano and carbonyl groups, with vitrimer‐CC exhibiting the highest surface potential, followed by V_1‐2_, and V_2‐1_ having the lowest. Furthermore, to visualize the charge distribution generated during contact between water droplets and the CCCN‐vitrimer, we observed the spreading process of water droplets on the vitrimer surface and measured its surface potential. Figure 2b shows sequential high‐speed camera images of a water droplet spreading across the CCCN‐vitrimer surface, illustrating rapid dispersion within 19 ms. It is noteworthy that due to the presence of imine groups, the surface of vitrimer exhibits hydrophobic properties,^[^ ^25^ ^]^ with a contact angle of approximately 94° (Figure S12). As a result, water droplets in contact with the vitrimer will quickly slide off the surface, exhibiting a rapid contact–separation dynamic behavior, which leads to the generation of triboelectricity.^[^ ^26^ ^]^ We then used a kelvin probe to test the surface potential distribution of the CCCN‐vitrimer, as shown in Figure 2c. Due to the accumulation effect of triboelectric charges, the surface potential of CCCN‐vitrimer increases with the number of droplet impacts. Furthermore, as water is a common triboelectrically positive material,^[^ ^27^ ^]^ the water droplets become positively charged upon triboelectrification. These positively charged droplets subsequently catalyze water oxidation, producing protons that participate in the CO_2_ reduction reaction (CO_2_RR).

We then calculated the electron distribution of the CCCN‐vitrimer, as shown in Figure 2d. The results indicate that electrons are primarily concentrated on the imine groups, confirming the electron‐rich effect induced by the presence of C═N bonds. To summarize, during the triboelectrification process with water droplets, the presence of cyano, carbonyl, and imine groups on the CCCN‐vitrimer membrane surface generates a large amount of frictional negative charge (electrons), which then accumulate around the imine groups. Given the high affinity of C═N bonds for CO_2_ adsorption, when CO_2_ interacts with the CCCN‐vitrimer, electron transfer occurs from the C═N bonds to the CO_2_ molecules. To verify the electron transfer mechanism between CO_2_ and the CCCN‐vitrimer upon contact, we conducted DFT calculations to analyze the charge distribution near the CCCN‐vitrimer surface, as shown in Figure 2e. When the distance between the CO_2_ molecule and the C═N bond exceeds 5 Å, no significant interaction is observed between the charges of CO_2_ and the nitrogen atom. However, as the distance decreases to 4 Å, electron accumulation occurs between the carbon atom of CO_2_ and the nitrogen atom of the C═N bond. This accumulation intensifies as the CO_2_ molecule moves closer to the nitrogen atom, leading to a significant build‐up of electrons. Consequently, when CO_2_ comes into contact with the C═N bond, electrons can rapidly transfer from the nitrogen atom to the CO_2_ molecule. To illustrate the catalytic process, we present a macroscopic schematic of the reaction mechanism following the impact of a water droplet on the CCCN‐vitrimer in the presence of CO_2_ (Figure 2f). First, CO_2_ is adsorbed onto the C═N groups (Figure 2fi). Then, as a water droplet contacts the CCCN‐vitrimer, triboelectric charging occurs, causing the CCCN‐vitrimer to become negatively charged, whereas the water molecules acquire a positive charge (Figure 2fii). Positive charges in the water drive oxidation reactions, producing protons that participate in the CO_2_RR. Simultaneously, electrons in the CCCN‐vitrimer are transferred to the adsorbed CO_2_, initiating a reduction reaction (Figure 2fiii). Finally, as the water droplet slides off the surface, the electrons in the CCCN‐vitrimer continue to facilitate CO₂RR, enabling the reaction to proceed continuously (Figure 2fiv).

### Performance of Contact‐Electroreduction of CO_2_

**Figure 3:**
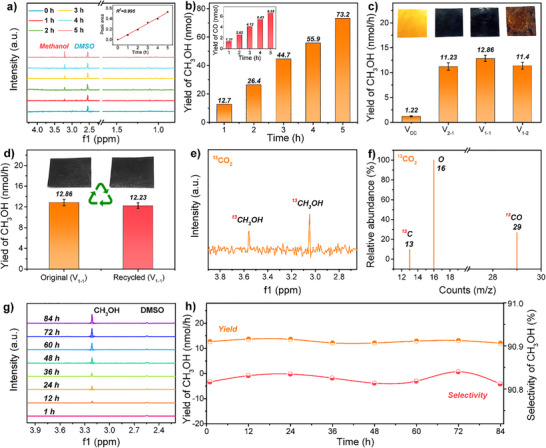
Performance of contact‐electroreduction of CO_2._ a) ¹H NMR spectra of the products from solid–liquid contact‐electrocatalytic CO_2_RR. The inset shows methanol peak area obtained based on the integration relative to the internal standard. b) Variation in methanol yield over time. The inset shows the corresponding variation in CO yield. c) Comparison of methanol yields in contact‐electrocatalytic CO_2_ reduction using CCCN‐vitrimers with different C═N bond contents and the CC‐polymer without C═N bonds. The inset shows optical photographs of these membranes. d) Comparison of methanol yields in contact‐electrocatalytic CO_2_ reduction using CCCN‐vitrimer before and after recycling. e) ^1^H NMR spectra of the contact‐electrocatalytic reaction solution using labeled ^13^CO_2_. f) GCMS of CO in the isotope experiment with ^13^CO_2_. g,h) ^1^H NMR, methanol yield and selectivity for CO_2_RR via contact electrocatalysis during 84‐h cyclic operation.

We subsequently investigated the performance of CO_2_ electroreduction upon contact between water droplets and the CCCN‐vitrimer. Before testing, the CCCN‐vitrimer was purged with N_2_ for 1 h on a heating plate at 110 °C to activate the C═N sites. The CCCN‐vitrimer was then attached to the inclined plate of a sealed chamber, where water droplets impacted the surface at a frequency of 1 Hz for 5 h. A small peristaltic pump was used to circulate the resulting aqueous solution, enriching the methanol concentration. Product samples were collected every hour and analyzed by ¹H NMR, with dimethyl sulfoxide (DMSO) as an internal standard and D_2_O as solvents. The results confirm the appearance of methanol at 3.2 ppm in the ¹H NMR spectrum, with the peak area increasing with reaction time (Figure 3a and the inset). The variation in methanol production over 5 h is shown in Figure 3b, whereas the inset illustrates the increase in CO production over time. Additionally, the CO yield, calculated based on GC analysis, is summarized in Table S2. The calculated methanol selectivity is approximately 91%. To evaluate the effect of C═N content on methanol yield during contact‐electroreduction of CO_2_, vitrimers with varying imine contents (CC, V_2‐1_, V_1‐1_, and V_1‐2_) were tested. The results show that the methanol yield from the CCCN‐vitrimers containing C═N is significantly higher than that from the C═N‐free polymer CC, indicating that the introduction of C═N enhances the contact electrocatalytic CO_2_RR. Furthermore, the methanol yield of V_2‐1_ with a lower C═N content is significantly lower than that of V_1‐1_ with a higher C═N content, further confirming that the presence of the C═N bond is essential for methanol production. In addition, V_1‐1_ produced slightly higher methanol yields than V_1‐2_. This may be attributed to the reduced content of cyano and carbonyl groups in the V_1‐2_ molecules, which leads to a decrease in contact electrification ability (Figure S17), thereby reducing the number of electrons involved in CO₂RR. To assess the recyclability of the CCCN‐vitrimer, V_1‐1_ was used as the study subject, and its methanol yield was compared before and after recycling (Figure 3d). The results demonstrate only a minimal change in methanol yield after recycling, confirming the excellent reusability of the CCCN‐vitrimer and its potential for practical applications.

To determine the origin of methanol and CO in the products, isotopically pure ^13^CO_2_ gas was used as the source in the solid–liquid contact electroreduction of CO_2_, and the products were analyzed by ¹H NMR and GCMS. When using ^13^CO_2_ instead of CO_2_, the ¹H NMR spectrum of the reaction solution in Figure 3e shows a doublet between 3.56 and 3.04 ppm, attributed to the protons coupled with ^13^C in ^13^CH_3_OH.^[^ ^24^ ^]^ This result directly indicates that CO_2_ serves as the carbon source for the solid–liquid contact electrocatalytic reduction of CO_2_ to CH_3_OH. Furthermore, the source of CO was investigated using GCMS. To enhance the detection sensitivity of the target compound, the selective ion monitoring (SIM) mode was employed to detect the presence of ^13^CO (*m/z* = 29) in the mixed gas. The mass spectrum (Figure 3f) reveals three main peaks for CO with the strongest signal at *m/z* = 29 corresponding to ^13^CO, and two additional fragments at *m/z* = 13 (^13^C) and *m/z* = 16 (O). The GC spectrum of gaseous products from contact electroreduction of CO_2_ using ^13^CO_2_ as the carbon source is shown in Figure S18. These findings clearly indicate that CO originates from contact electrocatalytic CO_2_RR. Furthermore, to demonstrate its excellent water resistance, CCCN‐vitrimer underwent a 7‐day soaking test, as shown in Figure S19. The results show that after 7 days, the CCCN‐vitrimer retained its surface morphology and flexibility, confirming its excellent water resistance and suitability as a durable solid–liquid triboelectric layer material for real‐world applications. Based on this, an 84‐h cyclic experiment was conducted to evaluate the stability of the solid–liquid contact‐electroreduction of CO_2_ to methanol. Liquid products were recirculated using a peristaltic pump to enrich methanol, with samples collected every 12 h for analysis via ¹H NMR and GC. The results show that the ^1^H NMR spectra of methanol over 84 h show a gradual increase in the peak area of methanol without the appearance of impurity peaks (Figure 3g), demonstrating the excellent stability of this method. Furthermore, after continuous operation for 84 h, the methanol yield remained at approximately 13 nmol·h^−1^ (Figure 3h), indicating that the CCCN‐vitrimer surface can continuously provide electrons for CO_2_RR under water droplet impact, achieving a selectivity for methanol exceeding 90%.

### Mechanism of Contact‐Electroreduction of CO_2_ to Methanol

**Figure 4:**
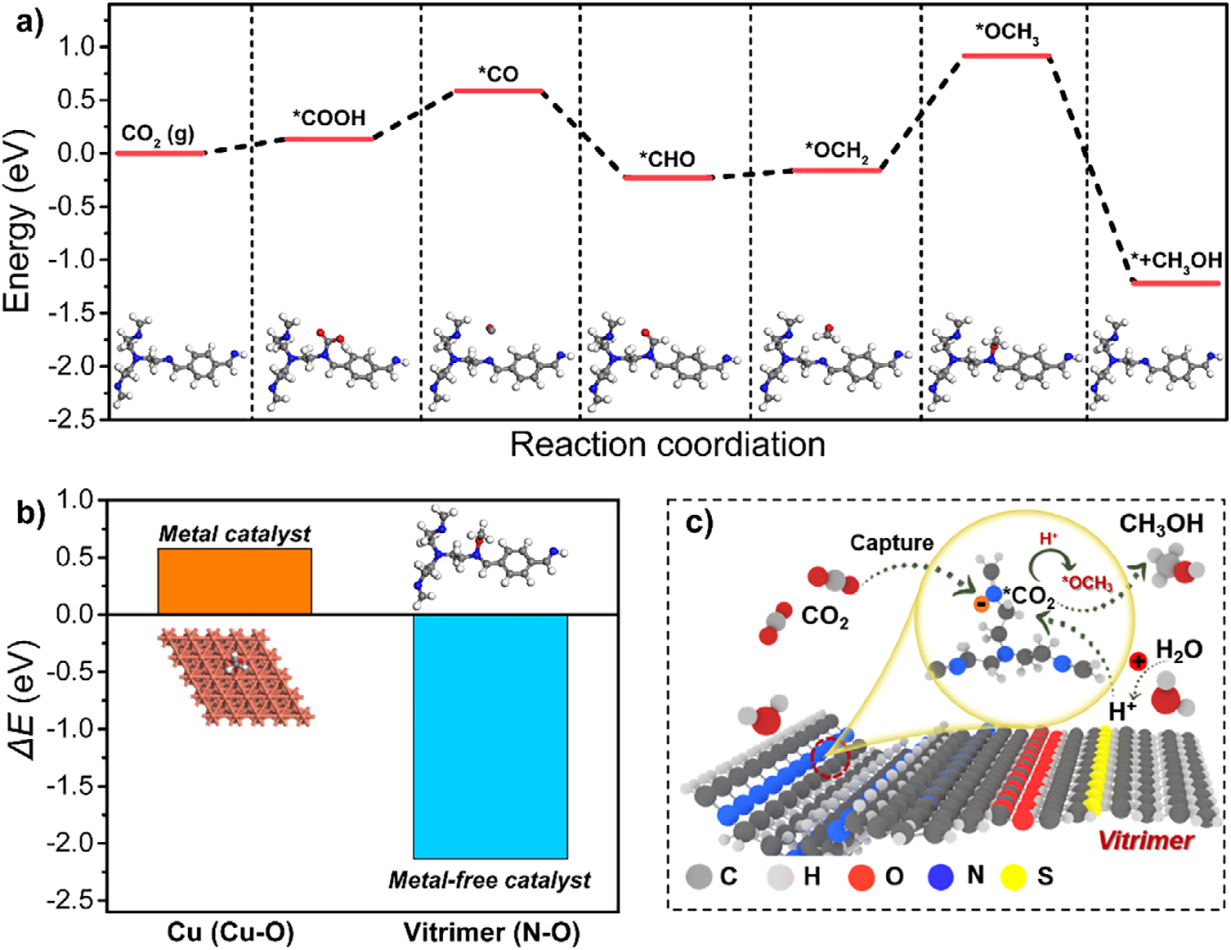
Theoretical calculations on contact‐electroreduction of CO_2_ to methanol mechanism. a) Energy diagrams of the methanol formation pathway on CCCN‐vitrimer. b) Calculated *ΔE* values for Cu─O and N─C bonds in the Cu–*OCH_3_ and N–*OCH_3_ intermediates on Cu (111) and CCCN‐vitrimer, respectively. c) Proposed synergetic mechanism for methanol formation from CO_2_ during the contact electrification of water droplets with CCCN‐vitrimer, mediated by imine bonds.

Based on the performance analysis of contact electrocatalytic CO_2_ reduction to methanol, density functional theory DFT calculations were employed to explore the underlying mechanism of CO_2_RR. As shown in Figure 4a, CO_2_ preferentially adsorbs onto the nitrogen site of the C═N bond in the initial activation step. At this site, the formation of *COOH can achieve a stable configuration with relatively low energy (0.13 eV), indicating that imine plays a synergistic role in facilitating CO_2_ activation. After *COOH is formed and subsequently reduced to *CO, the *CHO intermediate is generated. Due to the strong interaction between *CHO and the nitrogen atom, this step is exothermic. Subsequently, *CHO is adsorbed and further accepts electrons and protons to form *OCH_2_ and *OCH_3_, which are ultimately reduced to methanol. We found that the formation of *CH_2_O into *CH_3_O is highly endothermic and likely serves as the rate‐determining step (RDS) as this step requires endothermic energy (0.92 eV). Overall, these findings suggest that the presence of an imine functionality significantly enhances the electrocatalytic reduction of CO_2_to methanol.

To elucidate the distinctive reaction pathway of CCCN‐vitrimer from a molecular perspective, compared to traditional metal catalysts (e.g., Cu), the pathway for electrochemical CO_2_RR to methanol is common and involves the adsorption of the key reaction intermediate *OCH_3_ on a Cu atom (metal catalyst) or an N atom (metal‐free catalyst), forming Cu–*OCH_3_ or N–*OCH_3_. Subsequently, the Cu─O or N─O bond cleaves to produce *OCH_3_, as shown in Figure S20. To understand the high selectivity of the nonmetal catalyst in contact‐electrocatalytic CO_2_ reduction to methanol, we used *OCH3 as a probe to calculate the bond dissociation energies (*ΔE*) of Cu─O or N─O bond cleavage in the metal catalyst (Cu) and the nonmetal catalyst (CCCN‐vitrimer). The results show that on Cu (111), the cleavage of the Cu─O bond is an endothermic reaction, with a calculated *ΔE*_Cu‐O_ value of 0.57 eV (Figure 4b). In contrast, on the nitrogen atom of the C═N bond, the cleavage of the N─O bond is an exothermic reaction, with a calculated *ΔE*_N‐O_ value of −2.14 eV. This indicates that the cleavage of the N─O bond on the nonmetal catalyst CCCN‐vitrimer is more favorable for releasing *OCH₃, leading to a significant difference in methanol selectivity between the two catalysts. Therefore, based on catalytic performance, spectroscopic characterization, and DFT calculations, we propose a possible catalytic mechanism for the solid–liquid contact‐electrocatalytic reduction of CO_2_ to methanol on the nonmetallic CCCN‐vitrimer catalyst (Figure 4c). In the CCCN‐vitrimer molecule, the C═N bonds in the imine effectively capture and activate gaseous CO_2_. Simultaneously, contact electrification between CCCN‐vitrimer and water molecules leads to the oxidation of H_2_O, generating protons. In aqueous solution, these protons readily transfer to *CO_2_, and with the assistance of electrons, sequentially hydrogenate to form the *OCH_3_ intermediate. Because the cleavage of the N─O bond in C═N‐*OCH_3_ is exothermic (spontaneous), methanol is ultimately produced as the final product through subsequent hydrogenation steps.

## Conclusion

In conclusion, we have delineated a novel pathway for CO_2_ reduction to methanol that differs from traditional electrocatalysis. This approach uses a vitrimer‐based, metal‐free catalyst containing imine groups to catalyze CO_2_ reduction to methanol through static electricity generated by solid–liquid contact electrification, achieving an exceptionally high selectivity of 91%. The mechanism relies on the dual functionality of the imine bonds within the vitrimer, which not only provides excellent CO_2_ adsorption capacity but also creates an electron‐rich environment that facilitates the contact‐electrocatalytic CO_2_RR process. Additionally, compared to traditional metal catalysts, the N─O bond formed on the imine sites, which absorbs the key intermediate *OCH_3_ intermediate, is more readily cleaved to release methanol, thereby enhancing both product selectivity and yield. More importantly, the vitrimer catalyst exhibits excellent recyclability through C═C/C═N metathesis reactions, maintaining an essentially unchanged methanol production rate after multiple cycles. This reliability underscores its potential for practical applications. Overall, this sustainable and metal‐free strategy offers significant advantages over conventional catalytic methods, providing a promising pathway toward efficient methanol production and advancing CO_2_ utilization technologies.

## Supporting Information

The authors have cited additional references within the Supporting Information.

## Conflict of Interests

The authors declare no conflict of interest.

## Supporting information



Supporting information

## Data Availability

The data that support the findings of this study are available from the corresponding author upon reasonable request.
