# Supplementary material for: In Situ High Selectivity Contact‐Electroreduction of CO2 to Methanol Using an Imine‐Mediated Metal‐Free Vitrimer Catalyst
Source: Angew Chem Int Ed Engl. 2025 Mar 22;64(19):e202500222. doi: 10.1002/anie.202500222 (PMC12051734; doi:10.1002/anie.202500222)
Supplement: Supplementary file 1 — Supporting information [file ANIE-64-e202500222-s001.docx]

Supporting Information
©Wiley-VCH 2021
69451 Weinheim, Germany

***In Situ* High Selectivity Contact-Electroreduction of CO_2_ to Methanol Using an Imine-Mediated Metal-Free Vitrimer Catalyst**

Nannan Wang^[a] ‡^, Haisong Feng^[b]‡^, Jing Yang^[c]‡^, Jie Zheng^[a]^*, Yong-Wei Zhang^[c]^, Nikos Hadjichristidis^[d]^*, Zibiao Li^[a,e,f]^*

**Abstract:** Metal catalysts for the CO_2_ reduction reaction (CO_2_RR) face challenges such as high cost, limited durability and environmental impact. Although various structurally diverse and functional metal-free catalysts have been developed, they often suffer from slow kinetics, low selectivity, and non-recyclability, significantly limiting their practical applications. In this study, we introduce a recyclable non-metallic polymer material (vitrimer) as a catalyst for a new platform in contact-electro-catalysis. This approach harnesses the contact charges generated between water droplets and vitrimer to drive CO_2_RR, achieving methanol selectivity exceeding 90%. The imine groups within the vitrimer play a dual role, facilitating CO_2_ adsorption and enriching friction-generated electrons, thereby mediating efficient electron transfer between the imine groups and CO_2_ to promote CO_2_RR. After 84 h of CO_2_RR, the system achieved a methanol production rate of 13 nmol·h^-1^, demonstrating the excellent stability of the method. Moreover, the vitrimer retains its high-performance electrocatalytic activity even after recycling. Mechanistic studies reveal that, compared to traditional metal catalysts, the N-O bond in the imine, which adsorbs the key intermediate *OCH_3_, breaks more readily to produce methanol, resulting in enhanced product selectivity and yield. This efficient and environmentally friendly contact-electroreduction strategy for CO_2_ offers a promising pathway toward a circular carbon economy by leveraging natural water droplet-based contact-electrochemistry.

DOI:

**Table of Contents**

[**Experimental Procedures** 3](#_Toc190285764)

[1.Materials 3](#_Toc190285765)

[2.General characterization methods 3](#_Toc190285766)

[3. Instruments 3](#_Toc190285767)

[3.1 Nuclear magnetic resonance (NMR) spectroscopy 3](#_Toc190285768)

[3.2 Fourier transform infrared (FTIR) spectroscopy 3](#_Toc190285769)

[3.3 Thermal gravimetric analysis (TGA) 3](#_Toc190285770)

[3.4 Differential scanning calorimetry (DSC) 3](#_Toc190285771)

[3.5 Dynamic mechanical analysis (DMA) 3](#_Toc190285772)

[4. Synthetic Procedures 3](#_Toc190285773)

[4.1 Preparation of Polymers 4](#_Toc190285774)

[4.2 Reprocessing process 4](#_Toc190285775)

[5. Test Procedures 4](#_Toc190285776)

[5.1 Swelling ratio Test 4](#_Toc190285777)

[5.2 Gel Content Test 4](#_Toc190285778)

[6. Calculation of crosslinking density 4](#_Toc190285779)

[7. Calculation of activation energies (*E*_a_) in thermosets 4](#_Toc190285780)

[8. Contact electroreduction of CO_2_ assessments 4](#_Toc190285781)

[9. Density Functional Theory (DFT) calculations 5](#_Toc190285782)

[**Supporting Figures** 6](#_Toc190285783)

[**Supporting Tables** 13](#_Toc190285784)

[**References** 13](#_Toc190285785)

[**Author Contributions** 13](#_Toc190285786)

Experimental Procedures

**1.Materials**

Tris(2-aminoethyl)amine (TAA) (> 98.0%, TCI), terehthalaldehyde (TPA) (> 98.0%, TCI), benzene-1,3,5-tricarbaldehyde (BTA) (> 98.0%, TCI), allyl cyanoacetate (> 97.0%, TCI), L-proline (Aldrich), 2,2-dimethoxy-2-phenylacetophenone (99%, Aldrich), 1,6-hexanedithiol (> 97.0%, TCI), tetrahydrofuran (THF) (> 99.5%, TCI), acetonitrile (ACN) (> 99.5%, TCI), N,N-dimethylformamide (DMF) (≥ 99.8%, Aldrich), dichloromethane (DCM) (≥ 99.8%, Aldrich), hexane (> 99.5%, TCI), and ethanol (anhydrous, Aldrich) were used as received.

**2.General characterization methods**

Water droplets' spreading and retraction dynamics were recorded using a high-speed camera (Photron, FASTCAM Mini A). The surface potential of the impact location is measured using an electrostatic voltmeter with a non-contact Kelvin probe (TREK, Model 347). The probe is fixed on an XY-axis displacement stage, allowing continuous scanning of the surface according to a programmed path. By combining the coordinates, the distribution of surface potential on the surface can be obtained. The surface potential of CCCN-vitrimer with different C=N contents upon water droplet impact is measured using a surface electrostatic meter (KEYENCE-1000). The CO_2_ and N₂ adsorption performance of the material at ambient temperature and pressure was measured using an Autosorb IQ3 (Quantachrome Instruments, USA). An ion blower is used to blow the surface for approximately 10 s before each new experiment to eliminate excess. The products from CO_2_RR were analyzed using gas chromatography (GC) analysis. Gaseous products were collected from the flow gas, and 1 mL of the collected gas was analyzed by GC (8890, Agilent) equipped with FID and TCD detectors and argon (99.999%) as the carrier gas. Isotope measurements for CO_2_RR were performed on an Agilent 7890A GC-5975C MS. Selected ion monitoring (SIM) mode was used to detect the presence of ^13^CO (m/z=29) in the samples.

**3. Instruments**

3.1 Nuclear magnetic resonance (NMR) spectroscopy

The ^1^H NMR spectra were recorded on a JEOL ECAII 400 MHz, DMSO-*d6* and D_2_O were used as solvents for the NMR analysis, and the chemical shift was calibrated using residual undertreated solvents as the internal standards. General procedure for kinetics: A mixture of Kn1 (0.1 mmol) and A2 (0.1 mmol) was dissolved in 0.5 mL of DMSO-*d*_6_. The solution was then transferred into an NMR tube and sealed with a septum cap. Subsequently, the NMR tube was placed directly into the NMR machine, and measurements were initiated promptly. ^1^H NMR spectra were acquired at various temperatures, with readings taken at 300-second intervals, employing 8 scans per time point. The time elapsed between the initiation of the reaction and the recording of the first spectrum typically around 10 minutes. This duration was necessary for the locking and shimming processes of the NMR tube within the spectrometer probe. Product information was obtained through the integration of fixed areas corresponding to selected ^1^H NMR signals.

3.2 Fourier transform infrared (FTIR) spectroscopy

FTIR spectra were recorded with the High-Resolution FTIR (Bruker Vertex 80v) by attenuated total reflection (ATR) mode in the range of 4000- 400 cm^-1^. 32 scans were signal-averaged with a resolution of 4 cm^-1^ at room temperature.

3.3 Thermal gravimetric analysis (TGA)

TA Instrument Q500 was used to determine the initial thermal decomposition temperature (*T*_di_, temperature at 5% weight loss). About 10 mg of samples were placed into an alumina pan and heated from 50 to 800 °C with a ramping rate of 20 °C/min in nitrogen at a flow rate of 60 mL/min.

3.4 Differential scanning calorimetry (DSC)

The glass transition temperature (*T*_g_) of the specimen was characterized using TA Instruments Q100 calorimeter. About 10 mg of the sample was placed into an aluminium hermetic pan. The first heating scan was from 30 to 120 °C to erase any thermal history, followed by isothermal for 2 min, cooling to −20 °C, and subsequent heating (second heating scan) to 180 °C. The heating and cooling rate for the scans was 20 °C/min under a nitrogen flow of 50 mL/min. *T*_g_ was taken at the midpoint of heat capacity changes, while no melting behaviour was observed from the second heating curve.

3.5 Dynamic mechanical analysis (DMA)

Dynamic mechanical properties of the specimens were evaluated using TA Instrument Q800. The sample (20 mm (length) x 4 mm (width) x 0.15 mm (thickness)) was measured using a single cantilever mode at a frequency of 1 Hz, amplitude of 20 μm, and a temperature ramp of 3 °C/min. The storage modulus (E′) and tan delta (δ) were recorded as a function of temperature. Stress relaxation: samples were initially preloaded with a 0.1 N strain to maintain them straight. It was given 3 minutes to attain thermal equilibrium after reaching the testing temperature. Throughout the test, each specimen was subjected to a constant strain (1%), and the relaxation modulus was measured.

**4. Synthetic Procedures**

**4.1 Preparation of Polymers**

A standard procedure for synthesizing vitrimer featuring C=C/C=N, using the CCCN-vitrimer formulation (CC: CN = 1: 1) as an example, follows. A mixture of TPA (302 mg, 2.2566 mmol) and TAA (110.0 mg, 0.7522 mmol) in 10 mL THF was prepared and stirred for 2 min. Next, 2AC (451.4 mg, 1.1283 mmol) in 2 mL THF was added, and the resulting mixture was stirred at 50°C for 2 h. The solution was then transferred to a 5 cm-diameter PTFE petri dish and cured at 50°C for 12 h in a fume hood. Following that, the film underwent pre-curing under vacuum at 80 °C for 1 h and 120 °C for an additional 1 h. Post-curing was completed using a hot press (Collin P 200 PM) at 20 bar pressure, with a temperature profile of 160°C for 30 min. After annealing, a uniform film was obtained.

For the preparation of vitrimers with different C=C to C=N ratios (V2-1 and V1-2), the amounts of TPA, TAA, and 2AC were adjusted accordingly. This modification maintained the same procedure while varying the C=C to C=N ratio, enabling the synthesis of vitrimers with controlled C=C to C=N ratios.

For the preparation of vitrimer with C=C bonds (CC), a mixture of 2AC (1200 mg, 3 mmol), BTA (324.3 mg, 2 mmol), and THF (10 mL) was prepared, following the same process as outlined above.

**4.2 Reprocessing process**

The recyclable CCCN-vitrimer was cut into small pieces and sandwiched between two steel sheets lined with PTFE films. The assembly was then hot-pressed at 180°C under 30 bar of pressure for 30 min. After cooling to room temperature, a recycled film was obtained.

**5. Test Procedures**

5.1 Swelling ratio Test

For the swelling ratio test, approximately 100 mg of sample was weighed and recorded as m_0_ and then immersed in a sufficient amount of different organic solvents (such as DMF, DCM, THF, ACN, EtOH, and H_2_O) (approximately 10 mL) at 50 °C for 48 h. Then, the sample was removed and weighed as m_1_ after completely wiping the solvent on the surface with absorbent cotton. The swelling ratio was calculated using 100% × (m_1_ – m_0_)/mi.

5.2 Gel Content Test

For the gel content test, all samples (approximately 100 mg each) were individually placed into 20 mL vials containing DMF/DCM/THF/CAN/EtOH/ H_2_O for 48 h at 50 °C. Subsequently, they were washed three times with the same solvents before being dried in a vacuum oven at 80°C for 12 h. Here, m_0_ represents the initial mass, and m_1_ represents the final mass after drying. The gel content is calculated as 100% × m_1_/m_0_.

6. Calculation of crosslinking density

The crosslinking density (ρ) of the polymer network was calculated to be 3127 mol m^−3^ based on the E' value in the rubbery state, according to the following equation:^18^

$$\begin{aligned} \rho=\frac{E^{'}}{3RT}\#\left( 1 \right) \end{aligned}$$

where E′ refers to the storage modulus of **CCCN-vitrimer** network at (*T*_g_ + 40 °C), R and T represent the gas constant and absolute temperature, respectively.

7. Calculation of activation energies (*E*_a_) in thermosets

Activation energies (*E_a_*) were determined using the methodology reported in literature^19^.

The relaxation time refers to the time at which the relaxation modulus decreases to 1/e (~36.7 %) at different temperatures. The activation energy (*E_a_*) of the solid-state bond exchange was calculated via the Arrhenius equation (Eq. S2).

$$\begin{aligned} \tau^{*}\left( T \right)=\tau_{0}\exp\left( \frac{E_{a}}{RT} \right)\#\left( 2 \right) \end{aligned}$$

Eq. S2 can be transformed to Eq. S3

$$\begin{aligned} {\ln\tau}^{*}\left( T \right)=\ln\tau_{0}+\frac{E_{a}}{RT}\#\left( 3 \right) \end{aligned}$$

CCCN-vitrimer: ${\ln\tau}^{*}\left( T \right)=-14.85+7240/T$ $E_{a}=60 kJ /mol$

8. Contact electroreduction of CO_2_ assessments

The solid-liquid contact electroreduction of CO₂ experiment was conducted in a 2.5 L sealed chamber. The activated CCCN-vitrimer material was affixed to a 45° inclined plane inside the chamber. Before the reaction, the air in the sealed chamber was displaced with Ar gas, followed by introducing 130 mL of CO_2_ to maintain a CO_2_ concentration of approximately 5% in the chamber. Water droplets were introduced from above via a drip infusion system, with the droplets positioned 30 cm away from the inclined plane. A reservoir at the bottom of the sealed chamber collected the liquid products, which flowed out through an outlet. Driven by a small peristaltic pump, the liquid products were transported to the infusion system at the top for recirculation, enriching methanol. After the CO_2_RR experiments, the generated liquid products were detected using ^1^H-NMR spectroscopy, using DI water to dilute dimethyl sulfoxide (DMSO) by 200 times as an internal standard. In specific, after CO_2_RR experiments, a 450 μL product solution was acquired and mixed with 50 μL of D_2_O for the subsequent products quantification using ^1^H-NMR. Meanwhile, the gaseous products inside the sealed chamber were sampled with a 1 mL syringe and analyzed using GC to determine the CO yield.

9. Density Functional Theory (DFT) calculations

For the adsorption of CO_2_ and N_2_ and electron enrichment of Schiff bases, density functional calculations were performed using Gaussian 16^20^ at the B3LYP/def2svp level^21^. Additionally, the GD^22^ empirical dispersion correction were applied to accurately model the interaction between CO_2_ and CCCN-vitrimer. Furthermore, for the calculation of reaction pathways and bond dissociation energy, the first-principles calculations were performed using the Perdew-Burke-Ernzerhof (PBE)^23^ functional within the generalized gradient approximation (GGA) in the Vienna Ab initio Simulation Package (VASP 5.4.4)^24^. To account for van der Waals forces, the D3 dispersion correction with Becke-Johnson damping was incorporated in the DFT calculations^22^. The core-valence electron interaction was represented using the projector-augmented wave (PAW) method^25^. The structures were optimized until the forces acting on all atoms are below 0.02 eV Å−1 and the energies were below 10^–5^ eV, ensuring the convergence and reliability of the results. To ensure the accuracy and reliability of the DFT calculations, a cutoff energy of 500 eV was chosen for the plane-wave basis set. A Monkhorst-Pack k point of a gamma point is selected for structure optimization in the Brillouin zone.

Supporting Figures


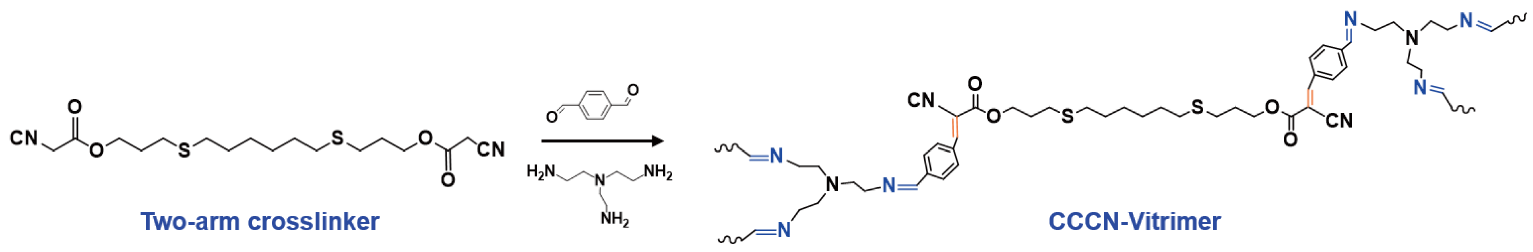


Figure S1. Schematic illustration of the preparation of vitrimer containing C=C and C=N bonds (CCCN-vitrimer).


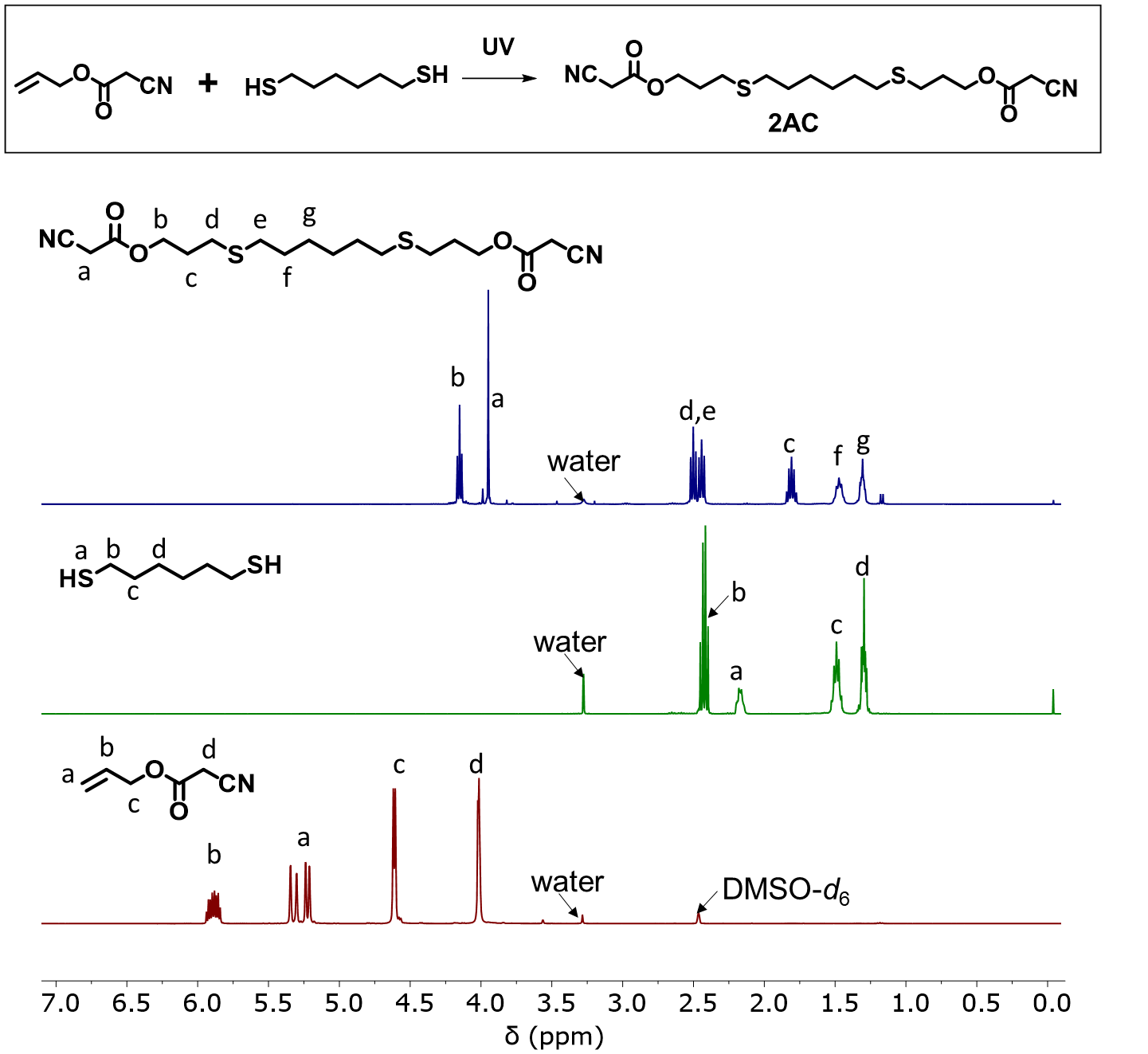


Figure S2. ^1^H-NMR spectra of the crosslinker (2AC) (DMSO-*d*_6_, 400 MHz, rt).


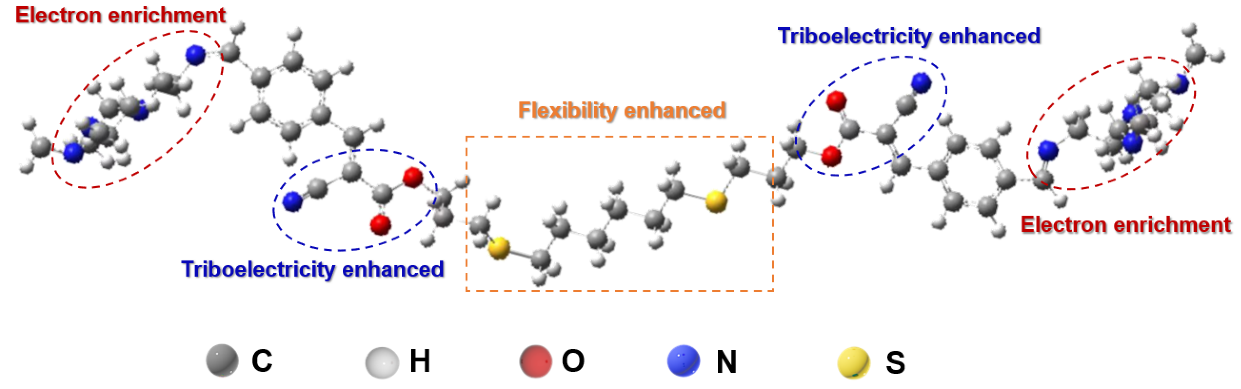


Figure S3. Schematic diagram of the functions of various structures in the CCCN-vitrimer molecule.


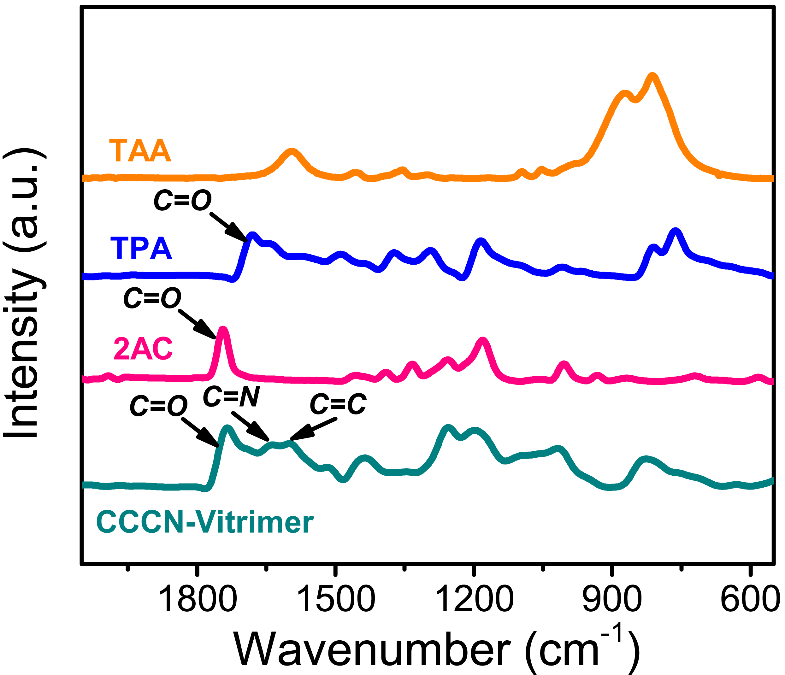


Figure S4. IR spectra of polymer monomers (TPA, TAA, and 2AC) and CCCN-vitrimer.


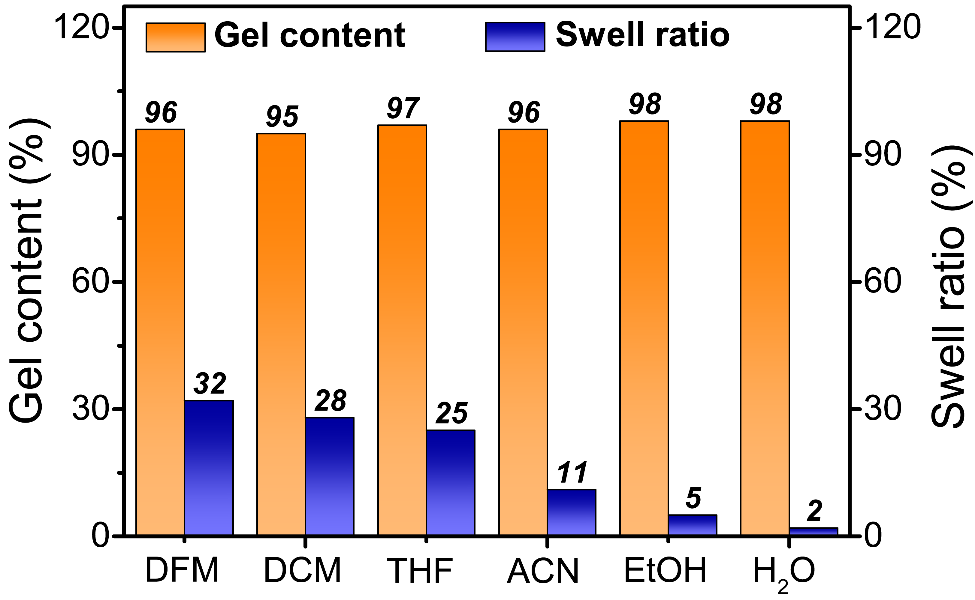


Figure S5. Swelling ratio and gel content of CCCN-vitrimer in various solvents.


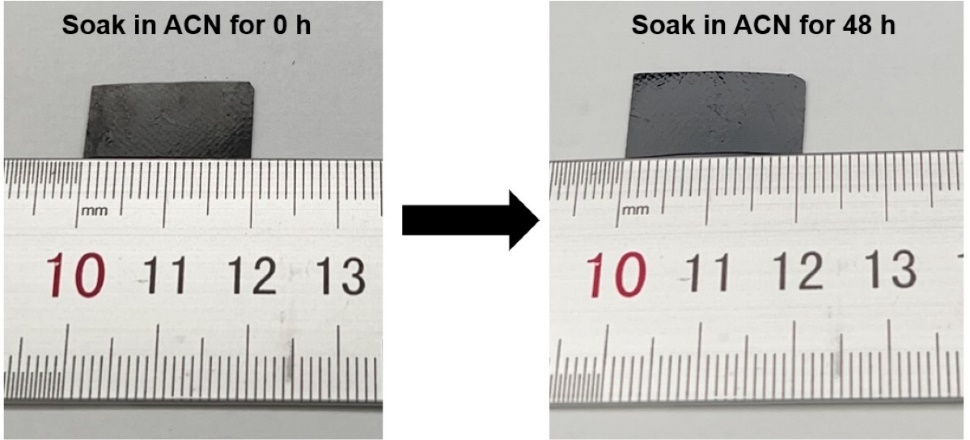


Figure S6. Photo of CCCN-vitrimer sample before and after soaking in ACN at 50 ^o^C for 48h.


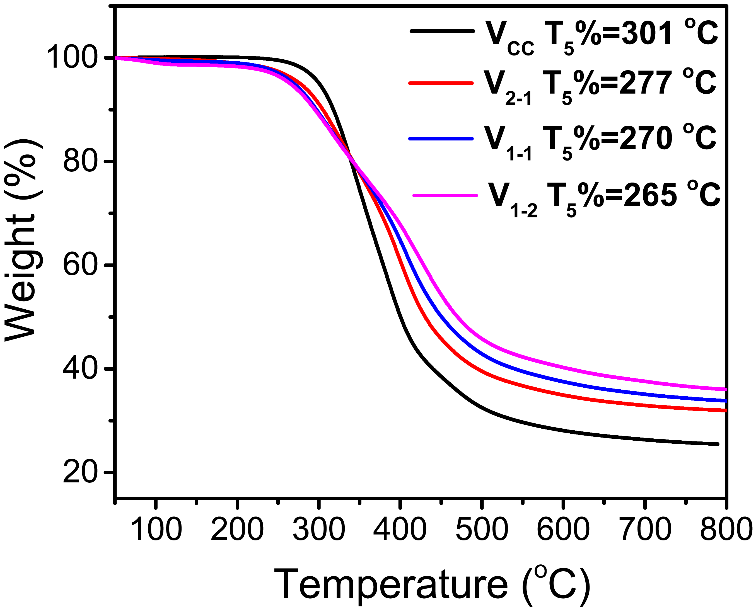


Figure S7. TGA curves of vitrimers (V_CC_, V_2-1_, V_1-1_, and V_1-2_).


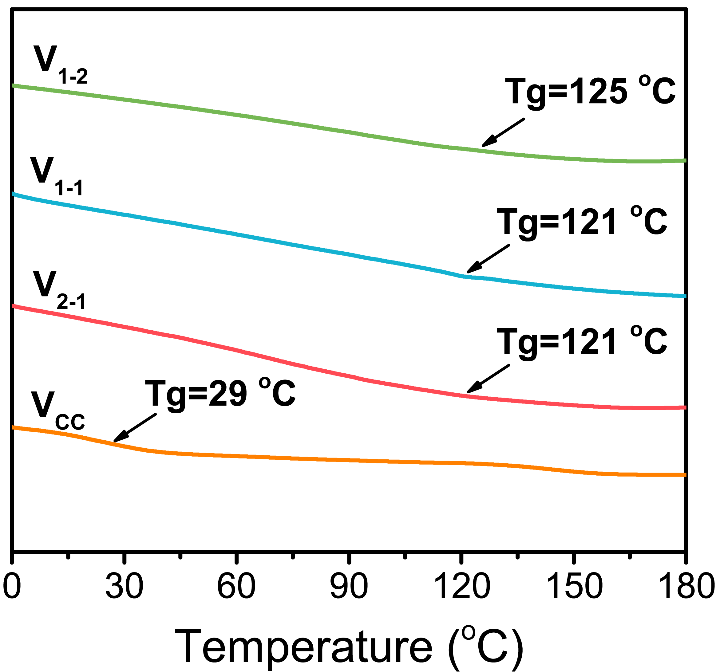


Figure S8. DSC curves of vitrimers (V_CC_, V_2-1_, V_1-1,_ and V_1-2_).


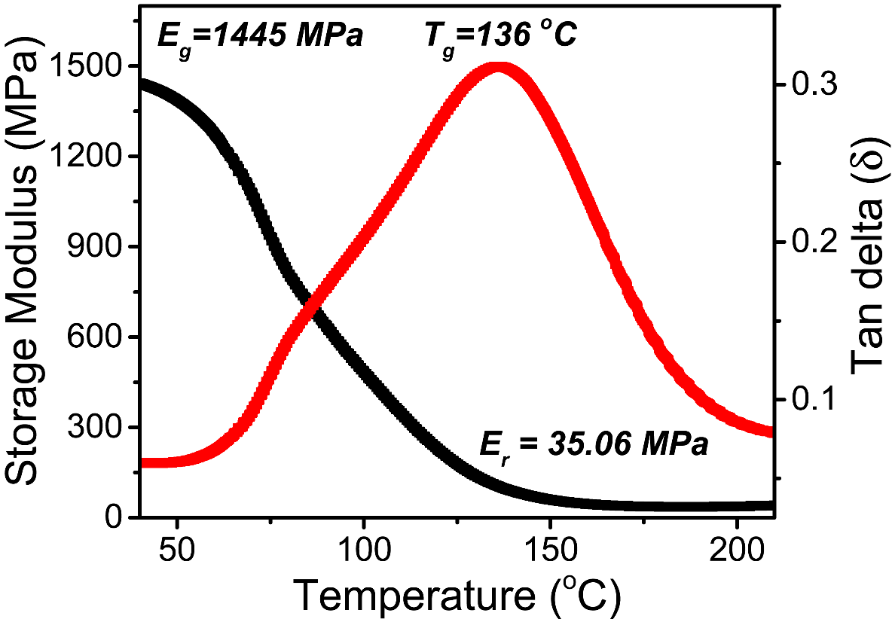


Figure S9. Temperature-dependent variations in storage modulus and tan delta for CCCN-vitrimer. E‘g and E‘r is storage modulus in the glassy state, and rubbery state, respectively.


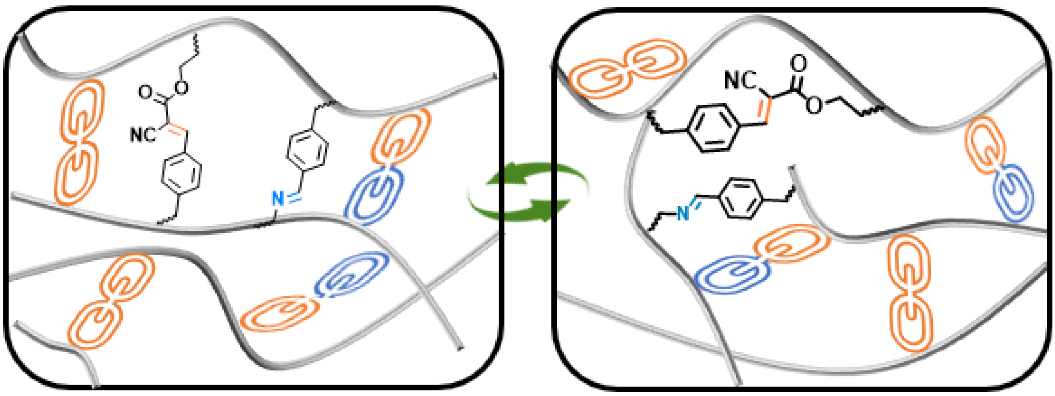


Figure S10. Schematic illustration of the dynamic structure of the vitrimer network showing recyclability.


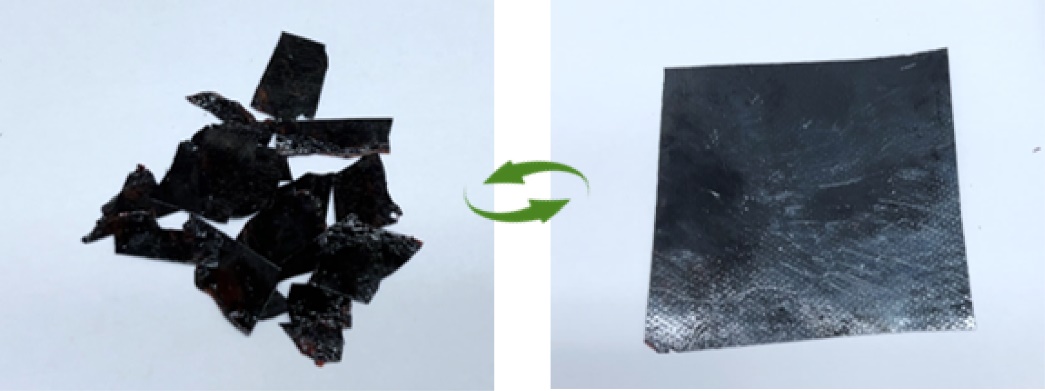


Figure S11. Photographs illustrating the recycling process of CCCN-vitrimer.


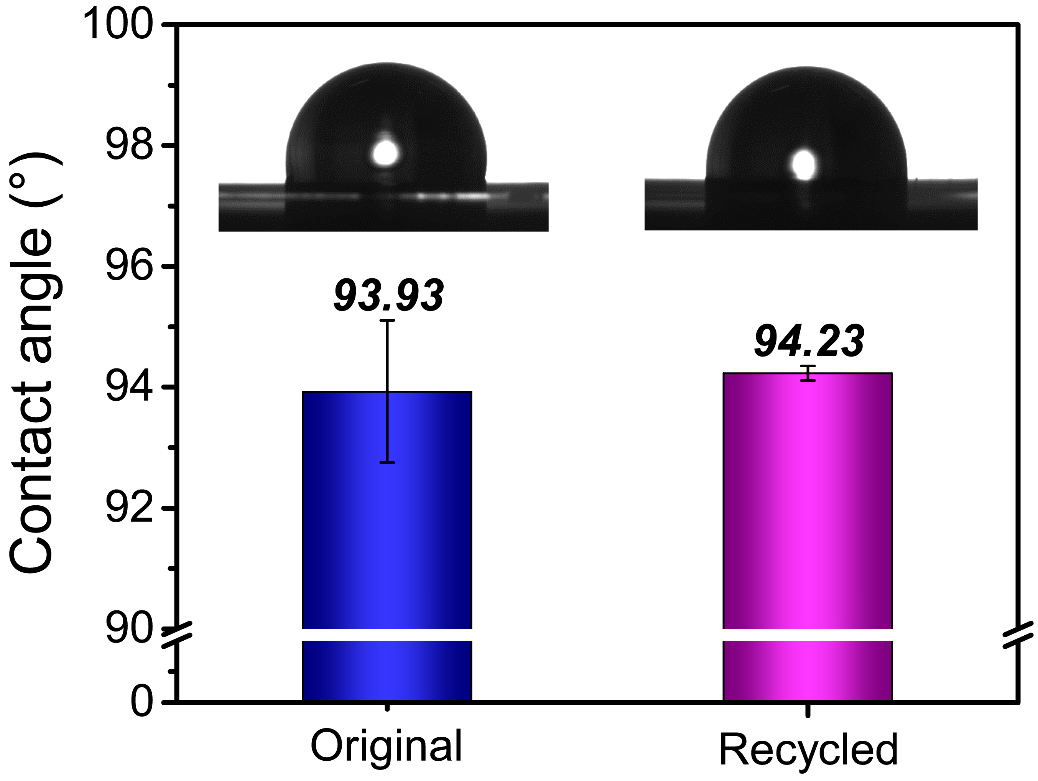


Figure S12. Comparison of contact angle of original and recycled CCCN-vitrimer.


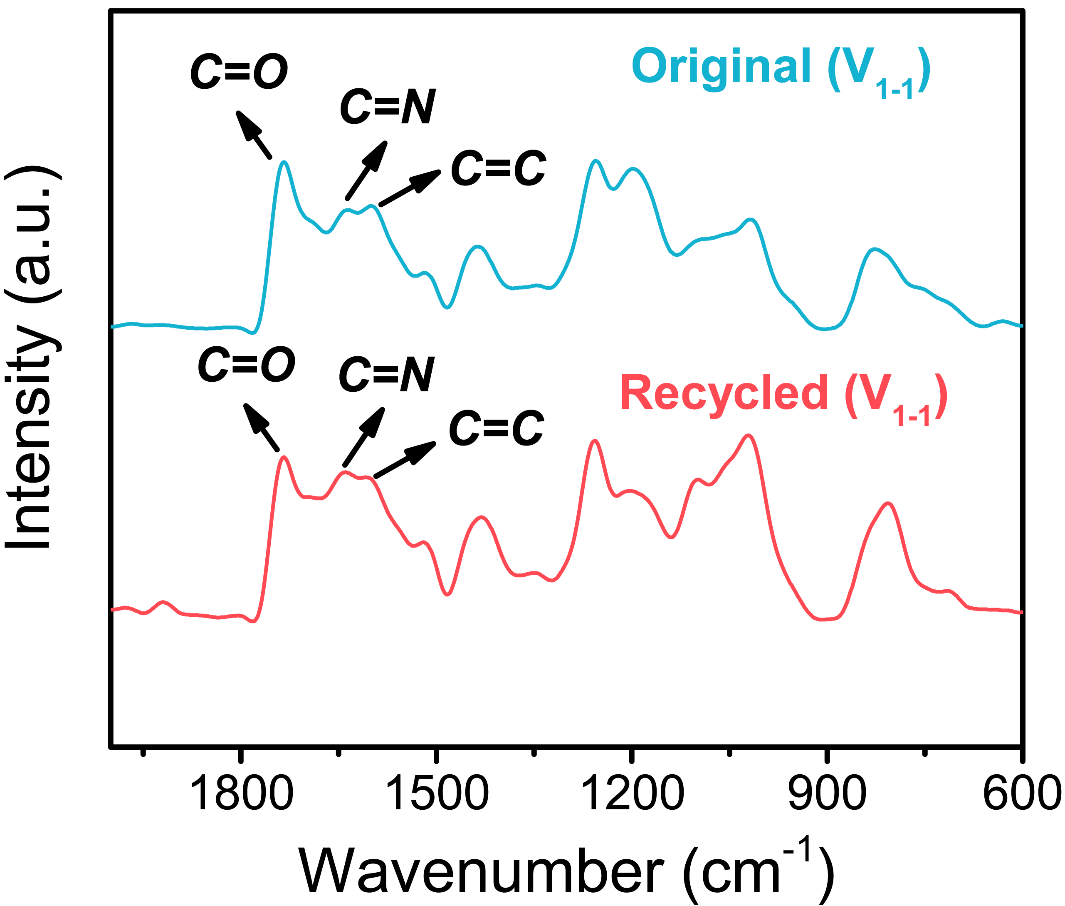


Figure S13. IR spectra of CCCN-vitrimer (V_1-1_) before and after recycling.


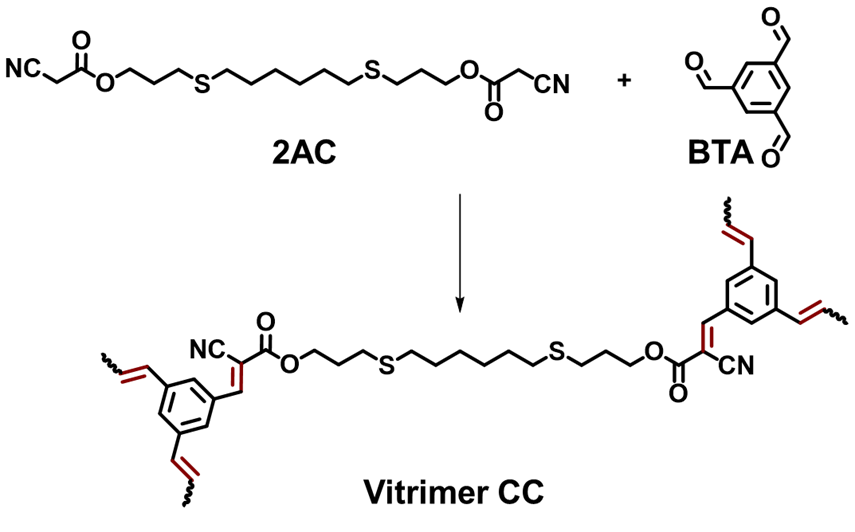


Figure S14. Schematic illustration for synthesis of polymer without C=N bonds (vitrimer CC).





Figure S15. IR spectra of vitrimers (V_CC_, V_2-1_, and V_1-2_).


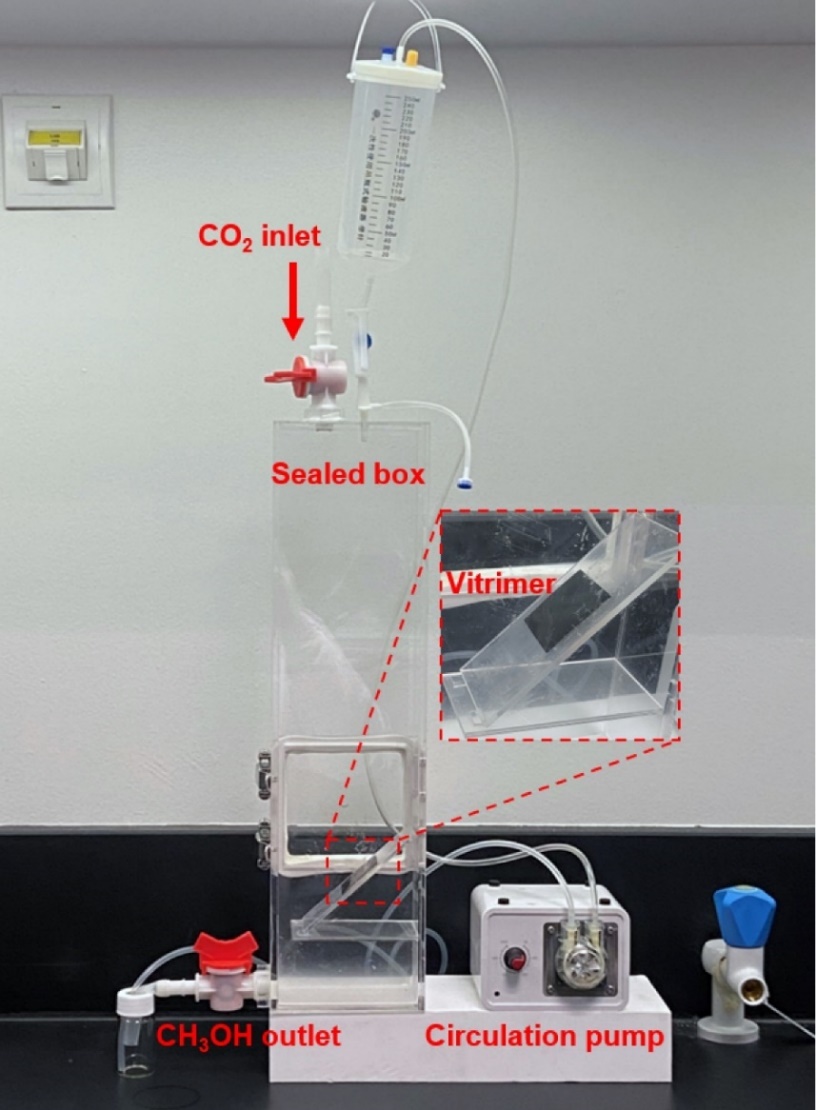


Figure S16. Optical photograph of the device for contact electroreduction of CO_2_ to methanol.


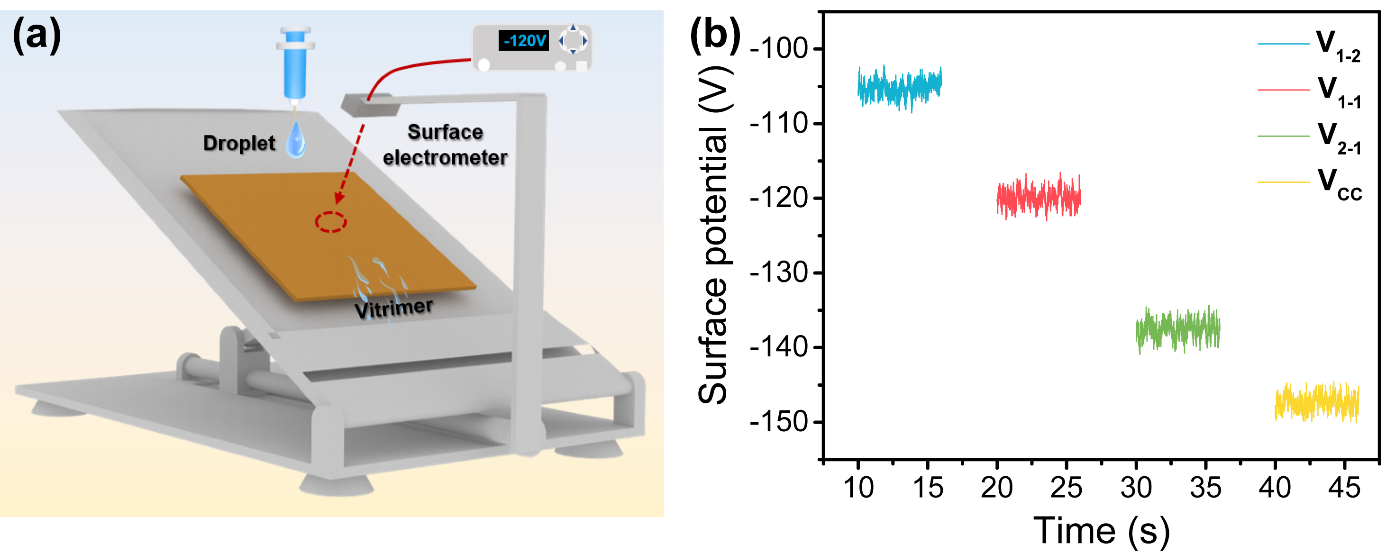


Figure S17. (a) Schematic illustration of surface potential measurement under water droplet impact on vitrimer. (b) Surface potential of vitrimer with different C=N contents measured by a surface electrometer.


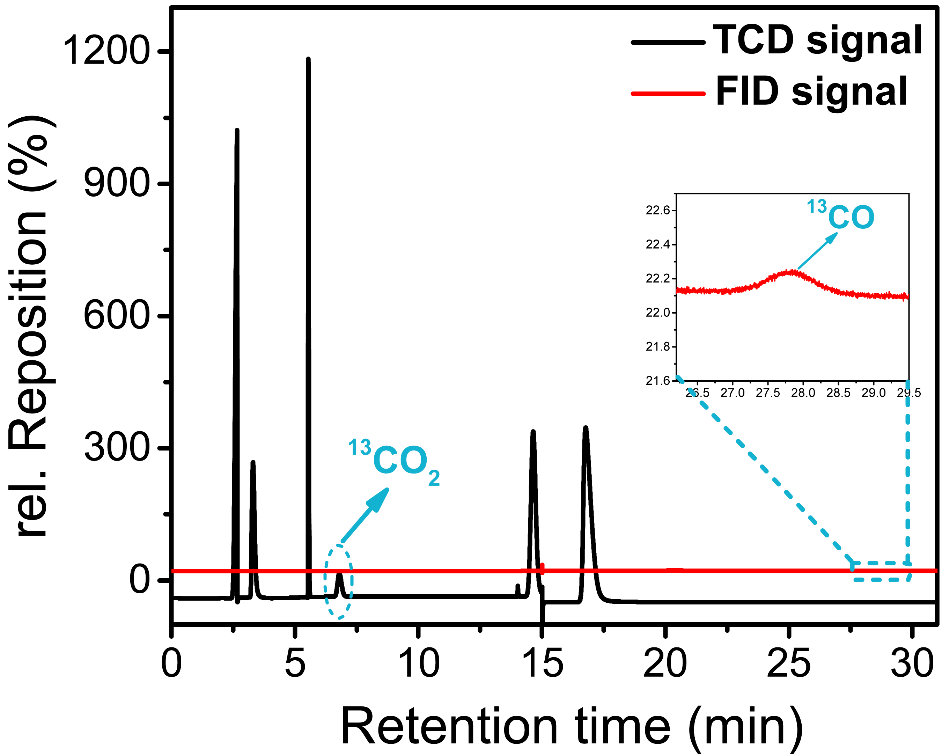


Figure S18. GC spectrum of gaseous products from contact electroreduction of CO_2_ using ^13^CO_2_ as the carbon source.

Figure S19.
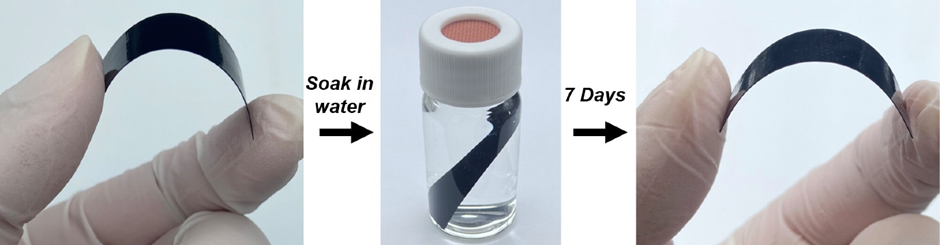
 Optical photograph of CCCN-vitrimer after soaking in water for 7 days.


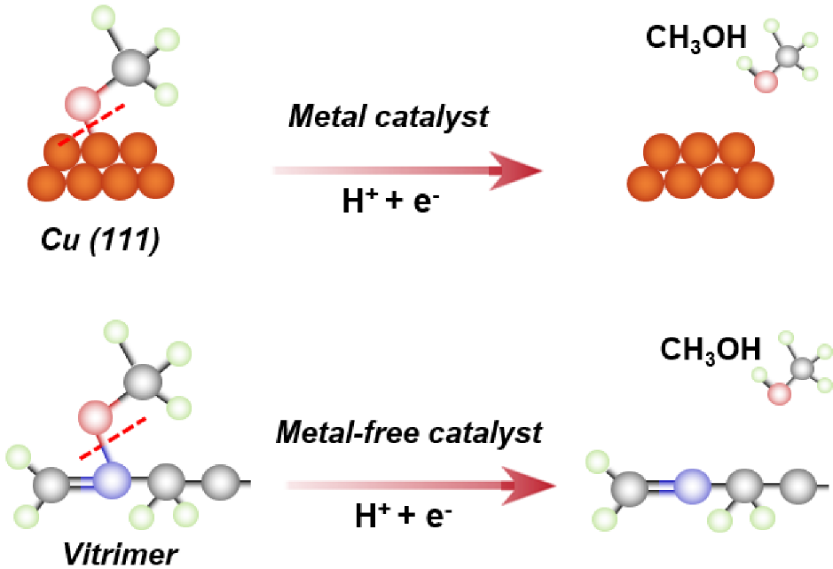


Figure S20. Schematic diagram of methanol formation through the cleavage of Cu–O and N–O bonds in a metal catalyst (Cu) and a non-metal catalyst (CCCN-vitrimer) adsorbed with the *OCH_3_ intermediate.

Supporting Tables

Table S1. Comparison of the adsorption energy of CO_2_ by Schiff bases of different materials.

| Schiff base materials | CO_2_ uptake (cm^3^/g STP) | Ref. |
| --- | --- | --- |
| Nitrogen-doped carbon aerogel | 26.7 | Li^26^ |
| tetraphenyladmantanebased poly (Schiff base) network | 50 | Li^27^ |
| Mesoporous Poly (Schiff-base) | 9.8 | Li^28^ |
| Schiff-base COFs | 65 | Zhang^29^ |
| Microporous Poly (Schiff Base) | 68 | Li^30^ |
| Schiff-Base COFs | 2.4 | Wang^31^ |
| CCCN-vitrimer | 1.7 | This Work |

**Table S2.** Calculation of CO yield from GC analysis.

| Sample | Injection volume/mL | Container volume/L | Time/h | Peak area | Yield/nmol |
| --- | --- | --- | --- | --- | --- |
| Standard | 0.25 | / | / | 426.26 | 2.23 |
| vitrimer | 1 | 2.5 | 5 | 0.5 | 6.55 |

References

[17] R. Gu, K. Flidrova, J.-M. Lehn, *Journal of the American Chemical Society* **2018**, *140*, 5560-5568.

[18] N. Zheng, G. Fang, Z. Cao, Q. Zhao, T. Xie, *Polymer Chemistry* **2015**, *6*, 3046-3053.

[19] Y. Nishimura, J. Chung, H. Muradyan, Z. Guan, *Journal of the American Chemical Society* **2017**, *139*, 14881-14884.

[20] M. Frisch, G. Trucks, H. Schlegel, G. Scuseria, M. Robb, J. Cheeseman, G. Scalmani, V. Barone, G. Petersson, H. Nakatsuji, *Wallingford, CT* **2016**.

[21] F. Weigend, R. Ahlrichs, *Physical Chemistry Chemical Physics* **2005**, *7*, 3297-3305.

[22] S. Grimme, J. Antony, S. Ehrlich, H. Krieg, *The Journal of chemical physics* **2010**, *132*.

[23] J. P. Perdew, K. Burke, M. Ernzerhof, *Physical review letters* **1996**, *77*, 3865.

[24] aG. Kresse, J. Furthmüller, *Computational materials science* **1996**, *6*, 15-50; bG. Kresse, J. Furthmüller, *Physical review B* **1996**, *54*, 11169.

[25] aG. Kresse, D. Joubert, *Physical review b* **1999**, *59*, 1758; bP. E. Blöchl, *Physical review B* **1994**, *50*, 17953.

[26] H. Li, J. Li, A. Thomas, Y. Liao, *Advanced Functional Materials* **2019**, *29*, 1904785.

[27] G. Li, B. Zhang, J. Yan, Z. Wang, *Chemical Communications* **2014**, *50*, 1897-1899.

[28] G. Li, B. Zhang, J. Yan, Z. Wang, *Journal of Materials Chemistry A* **2014**, *2*, 18881-18888.

[29] L. Zhang, R. Bu, X.-Y. Liu, P.-F. Mu, E.-Q. Gao, *Green Chemistry* **2021**, *23*, 7620-7629.

[30] G. Li, B. Zhang, Z. Wang, *Macromolecular rapid communications* **2014**, *35*, 971-975.

[31] Y. Wang, C. Kang, Z. Zhang, A. K. Usadi, D. C. Calabro, L. S. Baugh, Y. D. Yuan, D. Zhao, *ACS Sustainable Chemistry & Engineering* **2021**, *10*, 332-341.

Author Contributions

**Conceptualization:** N. W., J. Z., Z. L., N. H. **Investigation:** N. W., H. F. **Data curation:** N. W., J. Y. **Visualization:** N. W., J. Z. **Writing – original draft:** N. W. **Writing – review & editing:** N. W., Y. Z., Z. L., N.H. All authors discussed the results and commented on the manuscript.
